# Unexpected collateral impact after out of hospital resuscitation using LUCAS system

**DOI:** 10.1186/s13019-017-0643-z

**Published:** 2017-09-07

**Authors:** Jasmin Hasmik Shahinian, Jonas Quitt, Mark Wiese, Friedrich Eckstein, Oliver Reuthebuch

**Affiliations:** 1 Department of Cardiac Surgery, University Hospital Basel, Spitalstrasse 21, CH-4031 Basel, Switzerland; 2 Department of Anesthesiology, University Hospital Basel, Basel, Switzerland; 3 Department of Thoracic Surgery, University Hospital Basel, Basel, Switzerland

**Keywords:** Out-of-hospital resuscitation, LUCAS system, Flail chest, Emergency chest wall stabilization

## Abstract

**Background:**

Mechanical chest compression using a piston device during reanimation is often the only way to ensure stable chest compression at a constant rate and force. However, its use can be associated with severe fractures of the thoracic rib cage and endanger the clinical course of the patient. Thus, the usage of such a piston device during the reanimation has currently been classified as a mere Class IIB indication.

**Case presentation:**

We present a case of a 66-year-old male who underwent emergent CABG surgery after receiving out-of-hospital resuscitation as a result of myocardial infarction using the LUCAS system. Due to severe bilateral rib fractures a concomitant emergency chest-wall stabilization surgery had to be performed to ensure uncompromised graft flow to obtain stable cardiac function and hemodynamics.

**Conclusions:**

Reanimation using LUCAS-System might enable stable resuscitation conditions. However, it is crucial not to underestimate potential collateral damage which can in turn aggravate patient’s clinical condition.

## Background

Cardiac arrest followed by out-of-hospital resuscitation is a major public concern worldwide [1]. Detailed response mechanisms and protocols are stated in American and European guidelines to ensure correct management. Mechanical chest compression devices such as ‘The Lund University Cardiac Arrest System (LUCAS)’ or ‘Prehospital Randomised Assessment of a Mechanical Compression Device in Cardiac Arrest (PARAMEDIC)’ are so called piston devices, which are positioned over the sternum and compress the chest at a set rate and force. Both devices have been compared to the manual chest compression in PARAMEDIC and LINC trials [2, 3]. Neither of the trials could show a significant benefit from mechanical versus manual CPR (CardioPulmonary Resuscitation). Moreover, the time required for positioning the mechanical compression device prolongs the no-chest compression phase during resuscitation [4]. Hence, manual chest compression remains the standard of care in the resuscitation management of cardiac arrest. The use of mechanical compression devices remains a Class IIB recommendation being limited to special settings where accurate chest compressions cannot be delivered [4].

We present a case of a male patient after out-of-hospital resuscitation with the LUCAS System after cardiac arrest who underwent emergency CABG surgery. Due to the severe flail chest he experienced hemodynamic and respiratory instability with distinct ST-segment alterations caused by compromised graft flow. Subsequent chest wall stabilization could restitute myocardial perfusion and oxygenation.

## Case presentation

**Fig. 1:**
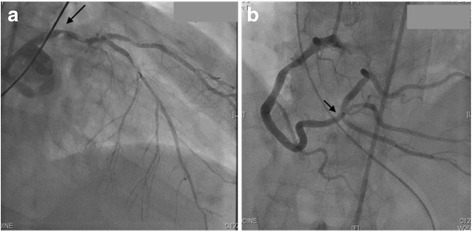
Coronary angiography; **a**) main stem stenosis (arrow), **b**) stenosis affecting PDA and RMA of RCA (arrow)

A 66-year-old male was brought to our emergency department via emergency rescue helicopter after out-of-hospital reanimation including defibrillation with AED (Automated External Defibrillator) and continuous mechanical chest compressions using the LUCAS CPR System. The patient was reanimated during 20 min until ROSC (Return of Spontaneous Circulation) occurred. The ECG (Electrocardiogram) performed on site showed signs of a STEMI (ST-Elevation Myocardial Infarction). An emergency coronary angiography was performed immediately after admission. It showed a high degree stenosis of the left main coronary artery, significant stenosis of the proximal LAD (Left Anterior Descending) and LCA (Left Circumflex Artery) with retrograde perfusion of marginal branches via right coronary artery (RCA). The RCA showed a severe stenosis at the level of the bifurcation affecting PDA (Posterior Descending Artery) and RMA (Right Marginal Artery) (Fig. 1a and b). With the indication for immediate surgical revascularization the patient was directly transferred to the operating room. Prior to surgery a seriously flailed chest with concomitant left-sided sternal fracture was noted after reanimation with LUCAS CPR system. The patient underwent on-pump coronary revascularization with arrested heart using blood cardioplegia. CABG surgery, aggravated by severe epicardial hematoma, was performed with following grafts: LIMA (left internal mammary artery) to LAD, vein jump-graft to diagonal and marginal branches as well as a vein jump-graft to PDA and RMA of the RCA. During LIMA-harvesting the extent of the left-sided rib fractures was noted.

**Fig. 2:**
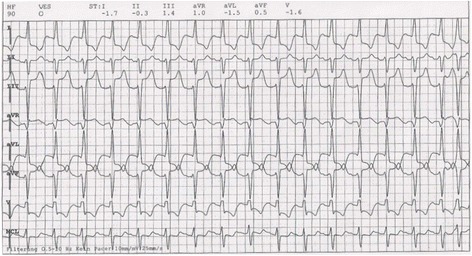
Intraoperative ECG; ST- elevations in inferior leads (III, aVF), ST – depression in lateral leads (I, aVL, V5)

For subsequent graft-flow measurement the TTFM (Transit Time Flow Measurement, MediStim, Norway) was applied. It showed flow (ml/min) as well as PI (Pulsatility Index) as follows: LIMA-LAD 26 ml/min, PI 2.5; vein jump-graft to diagonal and marginal branches of the left coronary system 111 ml/min, PI 1.5 and vein jump-graft to PDA and RMA 75 ml/min, PI 1.4. Under stable hemodynamics, CPB was discontinued. Moderate amounts of inotropes and vasopressors (milrinone 600mcg/h, adrenaline 2mcg/min and noradrenaline 2-4 mcg/min) were administered. During chest closure the ECG showed significant ST- elevations in inferior leads (III and aVF) and ST-depressions in I, aVL and V5 with a concomitant decrease of blood pressure and oxygenation (Fig. 2). Intraoperative TEE imaging showed slight hypokinesia of the left ventricle in correlation with the ST-deviations in ECG. A sternal retractor was placed again opening up the mediastinum to inspect the situs and to ensure stable hemodynamics. The flows of the grafts were re-evaluated showing stable results as detected previously. Upon the opening of the mediastinum the left ventricle showed relapsing good contractility which could be confirmed via TEE imaging. Upon the attempt to restore adequate intrathoracic space by manually repositioning the most prominent rib fractures patient’s blood pressure and oxygenation stabilized and improved. Hence, the hemodynamic instability was attributed to the flail chest noted preoperatively.

**Fig. 3:**
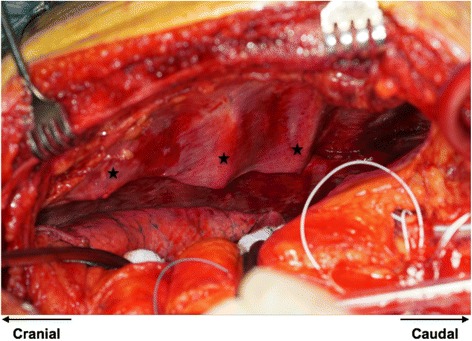
Left intrathoracic situs; multiple rib fractures (stars)

**Fig. 4:**
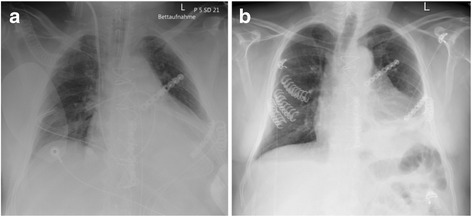
Chest X-ray showing chest wall reconstruction at the time of initial surgery (**a**) and 3 days postoperatively (**b**)

Multiple rib fractures limited intrathoracic space leading to compression of bypasses resulting in hemodynamic instability and ECG alterations as well as to increased intrathoracic pressure with subsequent deterioration of oxygenation (Fig. 3). The decision was made to perform an emergent chest-wall stabilization by repositioning the left sided multiple rib fractures prior to closing the chest to enable chest closure; thus, stabilizing the anatomic chest cavity and enabling stable cardiac function. This was performed by placing a Ripfix-Plate (MatrixRIB™ Fixation system, DePuy Synthes) on the 2nd, 5th rib and an osteosynthesis with clip (Stratos™, MedXpert) on the 9th rib (Fig. 4a), In addition, the left sided sternal fracture was fixed using a Ripfix-Plate. The procedure was performed via an initial submammary incision and further via a mini incision to access the 2nd rib over the pectoralis muscle at the point of the greatest instability. The fixation of the 2nd rib is generally not recommended especially if latero-dorsal instability is present, however this plays a minor role during parasternal instability. Further, we used only one screw during 2nd rib fixation instead of three due to the nature of the procedure as damage control, supine position of the patient, and limited ability to thoroughly assess the extent of the flail chest intraoperatively. Screw-fixation of the osseous part of the rib enabled absolute stability. Therefore, an additional fixation of the chondral part with the necessity of extending the incision was avoided. After fracture repositioning of the left thorax the sternum could be closed and the patient was transferred to the ICU.

Post-surgically the patient was hemodynamically stable and was about to be weaned. However, due to strong pain and paradox breathing during the weaning phase the indication for right-sided chest wall stabilization was given. The complete rib fracture repositioning involving the right thoracic wall was performed 3 days after the initial surgery using Stratos MedXpert clips on the 3rd to the 5th ribs (Fig. 4b). The intraoperative ST-alterations were resolved during the ICU stay with decreased peak levels of TroponinT (1559 ng/L to 897 ng/L) and CK-MB (75 mcg/l to 3.2mcg/l). The patient was transferred to the ward after 7 days of ICU stay.

## Discussion and conclusion

Mechanical reanimation with a piston device such as LUCAS, allows continuous mechanical chest compression during CPR [5]. Although trials using a porcine model could show improved systemic and coronary circulation [6, 7], large trials such as LINC and PARAMEDIC [2, 3] did not show significant differences for the secondary endpoints: ROSC, survival at 3 and 12 months and survival with favorable neurologic outcome. Overall, regarding the efficacy of the LUCAS device there is no evidence of superiority over manual CPR, currently remaining a class IIB recommendation with an evidence level (LOE) C [4]. Regarding the risk for injuries during reanimation, current literature reports higher frequency of collateral damage such as rib fractures, liver injury after mechanical compared to manual CPR, whereas the incidence of sternal fractures is higher [8, 9] during manual CPR. It is thus crucial not to underestimate potential injuries caused during CPR since these can foster adverse outcomes due to bleeding, hemodynamic instability, decreased intrathoracic volume restricting not only proper oxygenation of the lungs but also affecting cardiac perfusion by preventing myocardial perfusion and reducing cardiac filling during diastole. Our case shows an example of the severe consequences after mechanical reanimation with the LUCAS System. In our patient the fragments of the fractures were protruding into the thoracic cavity reducing the intrathoracic volume resulting in increased intrathoracic pressure and reduced myocardial perfusion. Hence the reduced perfusion of the grafts caused the ECG alterations observed perioperatively during chest closure. While the LUCAS CPR System is a recognized means to perform mechanical reanimation it is crucial to acknowledge and treat the side injuries since these can have a significant effect not only on the outcome of surgery but also on the overall mortality and survival.

## Abbreviations

AED: Automated External Defibrillator
CABG: Coronary Artery Bypass Grafting
CPB: Cardiopulmonary Bypass
CPR: Cardiopulmonary Resuscitation
ECG: Electrocardiogram
ICU: Intensive Care Unit
LAD: Left Anterior Descending
LCA: Left Circumflex Artery
LIMA: Left Internal Mammary Artery
LOE: Level of Evidence
LUCAS: The Lund University Cardiac Arrest System
PDA: Posterior Descending Artery
PI: Pulsatility Index
RCA: Right Coronary Artery
RMA: Right Marginal Artery
ROSC: Return of Spontaneous Circulation
STEMI: ST-Elevation Myocardial Infarction
TEE: Transesophageal Echocardiography
TTFM: Transit Time Flow Measurement
